# Central Venous Catheter-Related Bloodstream Infection with* Kocuria kristinae* in a Patient with Propionic Acidemia

**DOI:** 10.1155/2017/1254175

**Published:** 2017-01-17

**Authors:** Masato Kimura, Eichiro Kawai, Hisao Yaoita, Natsuko Ichinoi, Osamu Sakamoto, Shigeo Kure

**Affiliations:** Department of Pediatrics, Tohoku University Graduate School of Medicine, Sendai, Miyagi 980-8574, Japan

## Abstract

*Kocuria kristinae* is a catalase-positive, coagulase-negative, Gram-positive coccus found in the environment and in normal skin and mucosa in humans; however, it is rarely isolated from clinical specimens and is considered a nonpathogenic bacterium. We describe a case of catheter-related bacteremia due to* K. kristinae* in a young adult with propionic acidemia undergoing periodic hemodialysis. The patient had a central venous catheter implanted for total parenteral nutrition approximately 6 months prior to the onset of symptoms because of repeated acute pancreatitis.* K. kristinae* was isolated from two sets of blood cultures collected from the catheter. Vancomycin followed by cefazolin for 16 days and 5-day ethanol lock therapy successfully eradicated the* K. kristinae* bacteremia. Although human infections with this organism appear to be rare and are sometimes considered to result from contamination, physicians should not underestimate its significance when it is isolated in clinical specimens.

## 1. Introduction

Patients with indwelling foreign materials are known to be at considerable risk of bloodstream infections. Early diagnoses and effective treatments for intravascular catheter-related infections are therefore crucial.* Kocuria* species are widely distributed in nature and can be found in normal skin and among oral cavity flora in humans and other animals [1, 2].* K. kristinae* was first described in 1974 [3] and is an uncommon pathogenic organism in humans. However, some case reports indicate its emergence as a significant human pathogen [4–8]. We report a case of central venous catheter-related bacteremia with* K. kristinae* and a review of the literature.

## 2. Case Report

A 31-year-old Japanese man with propionic acidemia undergoing total parenteral nutrition presented with a high fever (39.5°C) after routine hemodialysis at his regional hospital. Although laboratory investigations revealed a slight elevation (1.2 mg/dL) of C-reactive protein (CRP), he was referred to our university hospital because Gram-positive cocci were detected in blood cultures from the dialysis circuit. He had been undergoing periodic hemodialysis three times a week for 4 years and had received total parenteral nutrition (TPN) for 6 months because of repeated acute pancreatitis.

The patient was originally diagnosed with propionic acidemia following plasma and urinary amino acid analysis in the neonatal period. He developed normally after commencement of a low-protein diet supplemented with L-carnitine, but he has mild mental retardation. Immunological abnormalities were not diagnosed until admission. At the age of 27 years, he developed cardiomyopathy with a low ejection fraction (EF = 22.2%) and oliguria and was treated by cardiac resynchronization with defibrillation therapy (CRTD) and hemodialysis (HD). On admission, his height was 1.57 m, weight was 56.5 kg, and body temperature was 36.7°C, and he was fully conscious. There was no evidence of abnormalities in the cardiovascular and pulmonary systems. Blood samples for culture were obtained from one peripheral venipuncture site and one central venous catheter (CVC). They were analyzed by VITEK® MS system (bioMérieux S.A., Marcy-l'Étoile, France) and both of them were only identified with* K. kristinae* (blood culture from the dialysis circuit was also identified with* K. kristinae* in regional hospital). Laboratory data were normal except for increased CRP levels (4.4 mg/dL). The CVC was left in place, antibiotic therapy with vancomycin was started, and the CVC was locked with ethanol for 5 days. Vancomycin was changed to cefazolin after* K. kristinae* was found in both blood cultures and its susceptibility to antibiotics was reported (Table 1). Antibiotics were administered for 16 days and the patient was discharged without complications after two serial negative blood cultures from CVC on another day. There was no reinfection after 3 months without antibiotic medication.

## 3. Microbiology

Two sets of blood cultures were obtained from one peripheral venipuncture site and one CVC. The blood samples were added into BacT/Alert® FA Plus and FN Plus resin bottles (bioMérieux S.A., Marcy-l'Étoile, France) and cultured BacT/Alert 3D automated microbial detection systems (bioMérieux S.A., Marcy-l'Étoile, France). Specimens were cultured on chocolate agar plate and were processed by VITEK MS system (bioMérieux S.A., Marcy-l'Étoile, France). VITEK MS is an automated microbial identification system that uses matrix assisted laser desorption ionization time-of-flight mass spectrometry (MALDI-TOF MS) technology to provide identification of microorganisms. Antimicrobial susceptibility tests are performed by Microscan WalkAway 96 Plus System (Beckman Coulter, Inc., CA, USA) and used Microscan Pos MIC 3.3 J panel (Beckman Coulter, Inc., CA, USA). Final molecular identification of microorganism was not carried out.

## 4. Discussion

*Kocuria* species are catalase-positive, coagulase-negative, Gram-positive coccoid actinobacteria belonging to the Micrococcus family.* To date, twenty-two* species have been classified in the genus:* K. kristinae*,* K. rosea*,* K. varians*,* K. palustris*,* K. rhizophila*,* K. marina*,* K. aegyptia*, and others [1, 2]. They are widely distributed in nature and have been found in normal skin and among oral cavity flora in humans and other animals.* K. kristinae* was first described by Kloos et al. in 1974 [3] and was not considered to be a human pathogen until about a decade ago. However, some case reports indicate the emergence of* K. kristinae* as a significant human pathogen, mostly in immunocompromised hosts [4], in patients with continuous ambulatory peritoneal dialysis (CAPD) [5], and in central venous catheter infections [4, 6–8].

We reviewed the literature on central venous catheter-related bloodstream infections (CRBSIs) with* K. kristinae* from 1974 to 2015. We identified 14 cases (including 7 nosocomial infections) and investigated the clinical backgrounds (Table 2 [4, 6–8]). Apart from hospital-acquired infections, there was female predominance (males, 2; females, 5) and the mean age was 44 years (range: 2–89). Long-term catheters are reported to have a lower risk of bloodstream infections than short-term catheters and almost all catheters were long-term types (long-term, 6; peripheral, 1) [9]. The revised guidelines from the IDSA (Infectious Diseases Society of America) published in 2011 recommend combination therapy with antibiotic administration through the colonized catheter for 10–14 days and antibiotic lock therapy in cases of uncomplicated CRBSI [10]. A review of the literature indicated that only one case was treated with combination therapy and the catheter was finally removed [4]. To the best of our knowledge, our patient represents the first successful treatment using combination therapy with antibiotics and antibiotic lock therapy to preserve the central venous catheter.

In conclusion, we have described a case of* K. kristinae* bacteremia in a patient with a central venous catheter and reviewed the literature. Although* K. kristinae* infections in humans appear to be rare and the organism was previously considered harmless, medical practitioners should be aware of the significance of this little recognized pathogen when it is isolated in clinical specimens.

## Figures and Tables

**Table 1:** The results of antimicrobial susceptibility testing.

Antimicrobial agent	MIC (mg/L)
Ampicillin	≤0.12
Cefazolin	≤2
Cefotiam	≤2
Cefpirome	≤2
Clindamycin	≤0.5
Erythromycin	≤0.25
Gentamicin	≤1
Imipenem/cilastatin	≤1
Levofloxacin	1
Teicoplanin	≤2
Vancomycin	1

MIC, minimum inhibitory concentration.

**Table 2:** Published cases of central venous catheter-related bloodstream infections with* Kocuria kristinae*.

Year	Age	Sex	Type of catheter	Removal of catheter	Medical condition or underlying disease	Treatment regimens	Reference
2002	51	F	Long-term	Yes	Ovarian cancer, three times recurrence	Meropenem, glycopeptide-ciprofloxacin, clindamycin	[4]

2010	89	F	Long-term	Yes	Infective endocarditis, short bowel syndrome	Vancomycin → teicoplanin → oxacillin	[6]
	37	F	Long-term	Yes	Gastric cancer	Piperacillin + tazobactam → ciprofloxacin	[6]
	2	M	Long-term	Yes	Congenital short bowel syndrome	Oxacillin + lock therapy with vancomycin	[6]
	68	F	Long-term	Yes	Gastric cancer	Oxacillin	[6]

2011	29	F	Peripheral	Yes	Pregnancy	Vancomycin + clindamycin	[7]

2015	1 m	M	Peripheral	Yes	Prematurity, healthcare-associated	Vancomycin, ceftazidime	[8]
	0.6 m	M	Peripheral	Yes	Prematurity, healthcare-associated	Vancomycin	[8]
	0.7 m	F	Peripheral	Yes	Prematurity, healthcare-associated	Vancomycin, ceftazidime	[8]
	1	F	Peripheral	Yes	Prematurity, healthcare-associated	Vancomycin, oxacillin	[8]
	0.6 m	F	Peripheral	Yes	Prematurity, healthcare-associated	Vancomycin, cefotaxime	[8]
	0.6 m	F	Peripheral	Yes	Prematurity, healthcare-associated	Vancomycin, cefotaxime	[8]
	2 m	F	Long-term	Yes	Leukemia, healthcare-associated	Vancomycin, piperacillin/tazobactam	[8]
